# *Exabundanti cautela*: From the tragedy of inadvertent sarcoma morcellation to inappropriate myoma screening

**DOI:** 10.1007/s10397-016-0932-x

**Published:** 2016-02-06

**Authors:** Thierry Van den Bosch

**Affiliations:** Department of Obstetrics and Gynecology, University Hospitals KU Leuven, 3000 Leuven, Belgium; Department of Obstetrics and Gynecology, Regional Hospital RZ Tienen, 3300 Tienen, Belgium

The recent commotion about the risk of unexpected malignancy during myoma morcellation may have divergent consequences. Some are positive for future women’s health care, while others may be negative. On the positive side, the ‘sarcoma awareness’ will lead to improvements in the diagnosis of myometrial lesions including leiomyosarcoma and in the technical aspects of safer, ‘spill-free’ myoma removal. On the negative side, the fear of an unexpected sarcoma may lead to unnecessary, more invasive surgery and avoidance of minimal invasive surgery—out of an abundance of caution.

The turmoil about the unexpected sarcoma, echoed and amplified in the press worldwide, may lead patients and doctors to have all too much faith in the adage that ‘any fibroid is a sarcoma, until proven otherwise’. Although this precautionary principle should be integrated in the decision making of any gynecologist before proceeding with a myomectomy, it could also have perverse effects. The latter may especially be true when confronted with the incidental finding of a well-defined myometrial lesion [1] on ultrasound examination. Some might even contemplate the introduction of a ‘screening program’ for sarcoma—‘because they care for their patients’. Indeed, the (public) perception of a fibroid being a ‘potential sarcoma’ could lead to following fallacious reasoning as illustrated in Fig. 1. There is no doubt that a leiomyosarcoma is a very aggressive and often lethal disease. If the lesion is confined to the uterus, the 5-year survival is 50 % [2], but only 20 % in case of more extended disease [3]. The cumulative risk for developing a sarcoma is about 5/10,000 [4]. This means that in a cohort of 20,000 women, 10 will develop a sarcoma. Depending on the stage of the cancer at diagnosis, 5 to 8 of those 10 women will ultimately die of the disease. Figure 1 summarizes what would happen if, motivated by the high mortality rates and by the repetitive alarming press releases, a screening program for sarcoma was launched at age 50. Given the 50 % incidence of fibroids at age 50, and based on the adage that ‘any fibroid is a sarcoma, until proven otherwise’, half of the screened population would be ‘screen positive’. Because of the diagnostic unreliability to distinguish between a benign myoma and a malignant leiomyosarcoma (LMS), the safest way to perform a ‘spill-free tumor removal’ would be a total abdominal hysterectomy. One may hypothesize that at age 50, half of the sarcomas would be picked up and that this would be at an early stage, confined to the uterus. Half of these women would therefore be curable, while 2 to 3 women would still die from the sarcoma. However, an abdominal hysterectomy is not free of risk. A mortality rate for abdominal hysterectomy of 1.5/1000 has been reported [5]. This would mean that out of the 10,000 women undergoing a hysterectomy, 15 women would die due to the surgery. Finally, in the “screen negative” group of women at age 50, one may assume that 5 women would develop a sarcoma later in life. These would then be diagnosed in a later stage of the disease and 3 to 4 of these women would die of the disease. Based on this simulation, the mortality rate in the screened group would be between 10 and 11/10,000 versus 2.5 and 4/10,000 in the unscreened group. The ‘collateral damage’ of screening a group of 20,000 women would be 12 to 17 extra deaths.

**Fig. 1:**
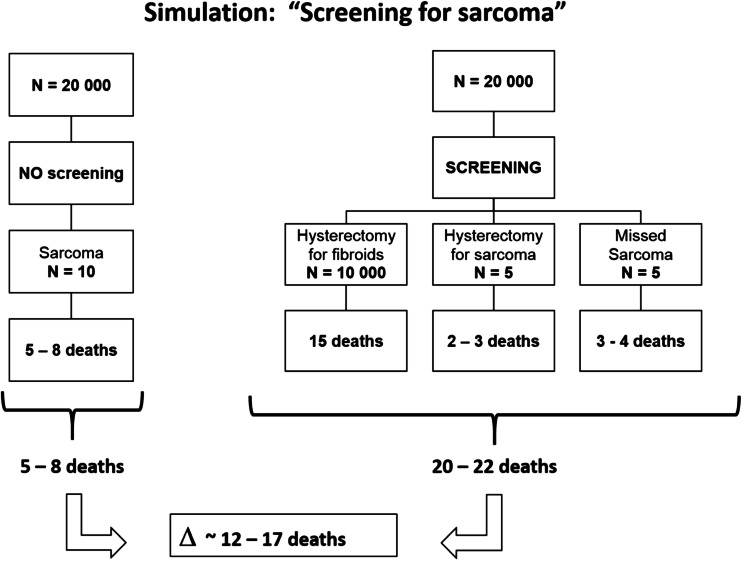
Simulation: ‘screening for sarcoma’

I acknowledge that this simulation based on a few selected publications is simplistic and that the figures are prone to bias. However, I mean it to illustrate the real danger of generalizing partial knowledge in medicine.

The risk of fibroid morcellation is an important women’s health issue and cannot be underestimated. However, it should be put in the right perspective as done by Brölmann et al. [6]. Both blunt denial of the problem and overreaction may have deleterious consequences.

The incidental diagnosis of a fibroid in an asymptomatic woman is rarely an indication for surgery.

## References

[1] Van den Bosch T, Dueholm M, Leone FP, Valentin L, Rasmussen CK, Votino A, Van Schoubroeck D, Landolfo C, Installé AJ, Guerriero S, Exacoustos C, Gordts S, Benacerraf B, D'Hooghe T, De Moor B, Brölmann H, Goldstein S, Epstein E, Bourne T, Timmerman D (2015). Terms and definitions for describing myometrial pathology using ultrasonography. Ultrasound Obstet Gynecol.

[2] Divakar H. (2008) Asymptomatic uterine fibroids. Best Pract Res Clin Obstet Gynaecol 22(4):643–654 10.1016/j.bpobgyn.2008.01.007 18375184

[3] Hacker NF, Berek JS, Hacker NF (2000). Uterine cancer. Practical gynecologic oncology.

[4] Amant F, Coosemans A, Debiec-Rychter M, Timmerman D, Vergote I (2009). Clinical management of uterine sarcomas. Lancet Oncol.

[5] Varol N, Healey M, Tang P, Sheehan P, Maher P, Hill D (2001). Ten-year review of hysterectomy morbidity and mortality: can we change direction?. Aust N Z J Obstet Gynaecol.

[6] Brölmann H, Tanos V, Grimbizis G, Ind T, Philips K, van den Bosch T, Sawalhe S, van den Haak L, Jansen FW, Pijnenborg J, Taran FA, Brucker S, Wattiez A, Campo R, O'Donovan P, de Wilde RL; European Society of Gynaecological Endoscopy (ESGE) steering committee on fibroid morcellation (2015). Options on fibroid morcellation: a literature review. Gynecol Surg.

